# Health policy regulations pertaining to advanced surgical devices—their socio-economic effects on ophthalmology practice

**DOI:** 10.1186/s13584-019-0286-8

**Published:** 2019-01-17

**Authors:** Dana Barequet, Aviad Tur-Sinai, Irit Barequet

**Affiliations:** 1 Department of Management, Faculty of Social Sciences, Bar-Ilan University, Ramat Gan, Israel; 2 Department of Economics and Management, The Max Stern Yezreel Valley College, 1930000 Yezreel Valley, Israel; 3 Goldschleger Eye Institute, Sheba Medical Center, Sackler Faulty of Medicine, Tel Aviv University, Tel Hashomer, Israel

**Keywords:** Astigmatism, Toric intraocular lenses, Cataract surgery, Out-of-pocket, Insurance coverage

## Abstract

The Israel Ministry of Health enacted regulations that aim to reduce private expenditure on healthcare services and mitigate social inequality. According to the modified rules, which went into effect in the second half of 2016, patients who undergo surgery in a private hospital and are covered by their healthcare provider’s supplemental insurance (SI) make only a basic co-payment.

The modified regulations limited the option of self-payment for advanced devices not covered by national health basket, meaning that patients for whom such devices are indicated had to pay privately for the entire procedure. These regulations applied to all medical and surgical devices not covered by national health insurance (NHI).

Toric intraocular lenses (IOLs) are a case in point. These advanced lenses are implanted during cataract surgery to correct corneal astigmatism and, in indicated cases, obviate the need for complex eyeglasses postoperatively. Toric IOL implantation has been shown to be highly cost-effective in both economic and quality-of-life terms. Limitations of the use of these advanced IOLs threatened to increase social inequality.

In 2017, further adjustments of the regulations were made which enabled supplemental charges for these advanced IOLs, performed through the SI programs of the healthcare medical organizations (HMOs). Allowing additional payment for these lenses at a fixed pre-set price made it possible to apply a supplemental part of the insurance package to the surgery itself. In mid 2018 these IOLs were included without budget in the national health basket, allowing for self-payment for the additional cost in addition to the basic coverage for all patients with NHI.

This case study suggests that, in their efforts to enhance health care equity, policymakers may benefit if exercising due caution when limiting the extent to which SI programs can charge co-payments. This is because, when a service or product is not available via the basic NHI benefits package, limiting SI co-payments can sometimes result in a boomerang effect - leading to an increase in inequality rather than the sought-after decrease in inequality.

## Policy principles

Israel’s National Health Insurance Law (1995) assures universal access to a standard package (“basket”) of basic medical services for all residents via health-maintenance organizations (HMOs) and public hospitals. The national health basket is reviewed annually by a government committee which considers additions of new medical services and technologies.

Over the years since 1995, and particularly since the passage of the 1997 Economic Arrangements Law, the share of public budgets in funding healthcare fell markedly and that of private expenditure correspondingly grew significantly. Per-capita public health expenditure rose from USD 1097 in 2000 to USD 1403 in 2015—by about 1.7% on annual average. Private expenditure, in contrast, climbed concurrently from USD 601 to USD 872—an average annual growth rate of 2.8% (all values in 2010 prices) [1]. One of the main reasons for these changes is lengthy queuing in the public system and patients’ wish to be able to choose their surgeon [2].

As of 2014, 87% of the population had supplemental health insurance through their HMOs (the most common form of additional insurance) and 53% had private health insurance. Patients who took out supplemental insurance (SI) could undergo surgery in private settings as long as both the operating facility and the surgeon had a contract with the SI provider. In these cases, patients made a basic compulsory co-payment for the procedure and self-pay for any special devices used; the SI covered the rest.

In November 2015, the Israel Ministry of Health enacted regulations designed to reduce private expenditure on healthcare services. The modified rules (under the jurisdiction of the Ministry of Health) focus on the funding of various surgical procedures by the HMOs’ SI plans, including surgeons’ fees and operating-room charges [3]. As will be described below and as summarized in Table 1, the rules governing the financing of advanced surgical operations and the insertion of specialized devices have undergone 4 phases. Before the regulatory changes (phase 1), if patients required special devices not covered by the basic basket, they could cover the additional cost of the devices fully out of pocket and have the rest of the surgery covered in the manner described above (i.e. mostly by the SI program and partly via a co-payment). In contrast, under the modified regulations (as initially implemented in phase 2), the operating facility was no longer allowed to charge patients for any costs beyond the basic co-payment for surgery. Therefore, special devices were disallowed for use under SI, since no additional charges could be applied, and the operating facility could not afford the high prices of these devices. Over time, the applications of this reform set multiple ongoing changes in motion, as explained below and summarized in Table 1. The reform had a direct and major effect on the daily management of patients who sought treatment under the coverage of their SI.

**Table 1:** How the financing of toric devices and their implantation has changed over time

Phase	Time period	Coverage in NHI basic basket	Coverage in supplemental insurance programs	Consequences
1	1995 to mid-2016	None^a^	Basic device – largely covered by SI, with small patient co-payment Toric device – fully covered by patient	The acquisition and implantation of toric devices for patients with supplemental insurance involved costs limited to co-payment for surgery and self-payment for the toric device.
2	Mid-2016 – mid-2017	None^a^	Basic device – largely covered by SI, with small patient co-payment Toric device – SI not allowed to charge patients; device to be fully covered by SI	SI programs ceased to include the option of self-coverage of toric devices and their implantation as these ceased to be financially feasible for them.
3	Mid-2017 – mid-2018	None^a^	Basic device – largely covered by SI, with either none or small patient co-payment Toric device – permitted along with the basic package with an additional code allowing for self-pay^b^	SI programs reinstated coverage of toric devices and their implantation. The acquisition and implantation of toric devices once again involved costs limited to co-payment for surgery and self-payment for the toric device
4	July 2018 and onward	Inclusion of toric device implantation with no funding of the toric lens itself^c^	Basic device – largely or fully covered by SI, with small patient with either none or small patient co-payment Toric device – operation covered by SI similar to basic device, with government-determined patient self-payment for the device. (Relevant for operations in private hospitals, with patient free to choose the surgeon and surgery in public hospitals covered by NHI)	Compared with phase 3, access to the new technology is accessible with self-payment for patients not possessing supplemental insurance.

^a^In phases 1–3 the NHI basic basket provided full coverage for cataract operations involving standard lenses but no coverage for operations involving toric lenses

^b^This change occurred gradually, through a series of government agreements with the various supplemental insurance programs

^c^In phase 4 patients with only basic NHI coverage have to cover the full cost of the toric lenses on an out-of-pocket basis

### Medical implications of the policy change

The modified regulations pertained to surgical devices in all areas of medical care that are not covered by national health insurance (NHI). Examples include novel meshes in laparoscopic hernia repair and advanced artificial tendons. Another example is the construction of individually fitted orthopedic joint prostheses (based on computed tomography reconstruction) as opposed to the basic option of ready-made prostheses. The use of the nano-knife, which destroys cancerous cells in tumors of the liver, kidney, or intestine without generating heat in surrounding healthy tissue, entails additional expense; thus, only the standard surgical procedure is covered in the basic basket. Yet another example is boost radiation for the unique treatment of single intraoperative radiation in breast carcinoma lumpectomy. It was previously self-paid; currently, these patients are covered for the surgical procedure only and need to pay out of pocket for several rounds of postoperative external radiation. Robotic surgery for intra-abdominal laparoscopies such as prostatectomy or hysterectomy, affording precision in the procedure and fast post-operative rehabilitation, is not covered by NHI; only regular laparoscopy is funded.

### Implications of the policy change for ophthalmology

Ophthalmology is a rapidly developing specialty that offers many advanced devices that are not included in the basic health basket, such as cataract surgery assisted by a femtosecond laser-.

We use cataract surgery as an important example that has immediate implications for the Israeli population. Cataract removal is the most common surgical procedure in current medical practice [4]. In its modern manifestation, spectacle independence (freedom from the postoperative need for corrective lenses) is becoming more and more important. Emmetropia (zero refractive error) can be achieved for patients who have myopic or hyperopic refractive errors by selecting the appropriate spherical intraocular lens power. However, 15–29% of patients who undergo cataract surgery have concomitant corneal or refractive astigmatism [5–7]. These patients are potential candidates for the implantation of toric intraocular lenses (IOLs). If a standard (monofocal) IOL is implanted in such patients at the time of their cataract surgery, their concomitant corneal astigmatism will limit their postoperative uncorrected visual acuity (UCVA) because standard IOLs correct spherical errors only. If the astigmatism component is left uncorrected at the time of the surgery, spectacle independence will be obviated.

The use of toric IOLs to correct corneal astigmatism is recommend for patients with regular corneal astigmatism who undergo cataract surgery and desire postoperative spectacle independence for distance vision [8, 9]. Previous research has analyzed this method and found it highly effective in correcting corneal astigmatism [10–13]. New toric IOLs effectively improve visual acuity, up to 93.3% of patients achieving UCVA of 20/40 or better in the affected eye following the procedure [10, 11, 13].

In recent years, it has been the European trend to allow co-payments for premium lenses and other advanced technologies while continuing to cover basic cataract surgery in full. In 2011, the Netherlands adopted new rules allowing patients to pay extra for multifocal lenses and the Czech Republic did the same by introducing co-payments for advanced technologies. Similar options were introduced in Germany and Turkey in 2012; in France and Sweden, co-payments have been allowed under some circumstances for some time [14].

In the United States, the Centers for Medicare and Medicaid Services ruled that astigmatism-correcting IOLs would not be fully reimbursed by Medicare [15]. Instead, Medicare reimburses for astigmatism-correcting lenses at the same level as it does for conventional IOLs and that patients interested in the astigmatism-correcting IOL would have to make up the difference.

Two studies have performed economic evaluations of toric IOL implantation versus monofocal IOL implantation during cataract surgery [16, 17]. Laurendeau et al. [15] estimated the lifetime costs of cataract surgery with bilateral toric or monofocal IOLs in patients with pre-existing corneal astigmatism in four European countries (France, Italy, Germany, and Spain). In this study, 70% of patients who received bilateral monofocal IOLs needed spectacles for distance vision, as against 26% of patients who were fitted with bilateral toric IOLs. The resulting saving for patients who receive toric IOLs depends on the cost of spectacles in each country, ranging from €308 in Spain to €693 in France. The study did not address the possible non-financial benefits of toric IOL implantation, such as patients’ visual functioning and health-related quality of life.

Pineda et al. [17] assessed the economic value of improved uncorrected visual acuity in patients with pre-existing corneal astigmatism and cataract treated with toric or monofocal IOLs in the U.S. They suggested that treating astigmatism with toric IOLs at the time of cataract removal yields several important benefits. Specifically, the typical patient who receives toric IOLs saves USD 35 in total costs relative to one who receives monofocal IOLs. These savings increase to USD 393 among patients who attain an UCVA of 20/25 or better. In addition, the cost per QALY (quality-adjusted life years, a measure of disease burden that combines quality and quantity of life) was found to be USD 349 lower with toric IOLs than with monofocal IOLs. Finally, toric IOLs were found to be more cost-effective than monofocal IOLs when combined with an intraoperative refractive correction such as limbal-relaxing incisions.

In Israel, no official records of intraocular lens implantation by types of lenses exist. A rough estimate of the percentage of the toric IOLs implanted annually, based on data provided voluntarily by Israeli surgical facilities, is 3–4% of all IOLs. Before the first stage of the reform, with toric IOLs not covered by NHI, patients with SI could benefit from these lenses at supplemental expense (estimated at USD 500–2000 per eye), with the rest of the surgery covered by the HMO’s SI. The healthcare policy regulations that went into effect in July 2016 revoked this right.

As noted above, the regulations allowed no charges above the patient’s basic co-payment. Therefore, no combination of basic coverage and additional charges for special devices was permitted. The novel lenses were so much more expensive than the standard ones that the operating facilities could not afford to use them when patients made only the basic co-payment. Patients who wished to have this type of IOL implanted during the cataract operation in order to avoid the need for astigmatism-correcting glasses had to cover out of pocket all facility charges plus the surgeon’s fee and the IOL (estimated at USD 3000–5000 per eye), with no reimbursement irrespective of their economic and financial resources. If they settled for the implantation of a regular (monofocal) IOL, to achieve clear vision they most likely had to be fitted postoperatively with eyeglasses that would address the residual astigmatism. Patients paid for these expensive glasses out-of-pocket, not to mention the dependency and burden of adjusting to the discomfort of these complex lenses.

Thus, the regulations aimed to mitigate social inequality by preventing additional surgical charges but, as initially implemented, they have amplified inequality in access to advanced medicine and therefore reduced consumer choice. Patients who could not afford completely private surgery were unable to benefit from a procedure that involved special devices, particularly toric IOLs, because they had to pay for all elements of the surgery and the toric IOL with no reimbursement.

However, in a second stage of the reform which was implemented in mid-2017 (phase 3), SI provided new codes for the novel IOLs and thus allowed the surgery to be performed under co-payments that varied according to the type of IOL implanted. The acquisition and implantation of toric devices once again involved only limited costs for patients with SI.

Finally, in an additional phase (phase 4), a decision was made in July 2018 to include IOLs and their implantation in the national health basket, but without additional funding from the government. Accordingly, as of July 2018, the cost of the operation itself is being covered by NHI in public hospitals for all patients (i.e. whether or not they have SI) and the patients may self-cover the cost of the special device. Thus, compared with phase 3, access to the new technology improved for patients not possessing SI. Patients with SI can continue to be able to undergo the operation in a private hospital, with choice of physician, with SI covering most of the costs of the operation and the special device (the toric IOL) is being self-paid at a fixed price.

## Summary and conclusions

This paper addresses several implications of Israel Ministry of Health healthcare policy regulations that went into effect in July 2016. Under the 2016 rules, patients are not charged for any costs of private surgery beyond the basic co-payment required by the HMO’s SI. The modified policy aimed to mitigate social inequality and improve access to medical treatments.

Before the reform, devices yet to be included in the Israeli national health basket were accessible by means of a supplemental payment to cover their cost, while the surgery itself was mostly covered (apart from the basic co-payment) by the HMO’s SI. The restrictions attending to the additional payment mitigated access to advanced devices. Afterwards, the rules were further modified through the HMOs’ SI programs to allow fixed charges for advanced devices in addition to basic coverage of the procedure.

We gave several examples of such novel devices that are used in diverse fields of surgery, and elaborated on the potential implications of the modified regulations for patients who need toric IOLs to correct astigmatism in cataract surgery. These advanced lenses are implanted during cataract surgery to correct corneal astigmatism. Toric IOL implantation has been shown to be highly cost-effective in both economic and quality-of-life terms [17]. Clinical studies on these lenses demonstrated excellent uncorrected distance visual outcomes and low residual refractive cylinder [8, 10, 11, 13]. Consequently, most patients who are fitted with bilateral toric IOLs achieve spectacle independence for distance vision. As mentioned, further adjustments of the regulations had to be made in order to make them affordable for most patients, attain the aims of the law, and enhance access to novel and necessary technologies. Inclusion in the package fee for the operation allowing additional payment for these lenses was finally introduced at a fixed preset price.

In sum, the 2016 healthcare policy reform was designed to lower private expenditure for healthcare services. Initial significant limitations led to adjustments in order to bridge this gap and enhance access to novel necessary technologies. This demonstrates the evolutionary process initiated to limit private expenditure that prevented access to novel devices such as the toric IOLs, and finally resulting in inclusion in the health basket, allowing additional self-cost for permitting the implantation of these IOLs as a supplement to the basic cataract surgery.

This case study suggests that, in their efforts to enhance health care equity, policymakers may benefit if exercising due caution before limiting the extent to which S) programs can charge co-payments. This is because, when a service or product is not available via the basic NHI benefits package, limiting SI co-payments can sometimes result in a boomerang effect - leading to an increase in inequality rather than the sought-after decrease in inequality.

## Abbreviations

HMO: Health maintenance organization
IOL: Intraocular lens
NHI: National health insurance
QALY: Quality adjusted life years
SI: Supplemental insurance
UCVA: Uncorrected visual acuity
